# The German Version of the Humor Styles Questionnaire: Psychometric Properties and Overlap With Other Styles of Humor

**DOI:** 10.5964/ejop.v12i3.1116

**Published:** 2016-08-19

**Authors:** Willibald Ruch, Sonja Heintz

**Affiliations:** a Department of Psychology, University of Zurich, Zurich, Switzerland; Department of Psychology, University of Western Ontario, London, Canada

**Keywords:** Humor Styles Questionnaire (HSQ), German adaptation, reliability, factorial validity, factorial invariance, styles of humorous conduct, comic styles

## Abstract

The Humor Styles Questionnaire (HSQ; Martin et al., 2003) is one of the most frequently used questionnaires in humor research and has been adapted to several languages. The HSQ measures four humor styles (affiliative, self-enhancing, aggressive, and self-defeating), which should be adaptive or potentially maladaptive to psychosocial well-being. The present study analyzes the internal consistency, factorial validity, and factorial invariance of the HSQ on the basis of several German-speaking samples combined (total N = 1,101). Separate analyses were conducted for gender (male/female), age groups (16–24, 25–35, >36 years old), and countries (Germany/Switzerland). Internal consistencies were good for the overall sample and the demographic subgroups (.80–.89), with lower values obtained for the aggressive scale (.66–.73). Principal components and confirmatory factor analyses mostly supported the four-factor structure of the HSQ. Weak factorial invariance was found across gender and age groups, while strong factorial invariance was supported across countries. Two subsamples also provided self-ratings on ten styles of humorous conduct (n = 344) and of eight comic styles (n = 285). The four HSQ scales showed small to large correlations to the styles of humorous conduct (-.54 to .65) and small to medium correlations to the comic styles (-.27 to .42). The HSQ shared on average 27.5–35.0% of the variance with the styles of humorous conduct and 13.0–15.0% of the variance with the comic styles. Thus–despite similar labels–these styles of humorous conduct and comic styles differed from the HSQ humor styles.

## Introduction

The Humor Styles Questionnaire (HSQ; Martin, Puhlik-Doris, Larsen, Gray, & Weir, 2003) assesses the four humor styles of affiliative, self-enhancing, aggressive, and self-defeating to allow for a multidimensional assessment of everyday functions of humor, with a focus on functions that are relevant for one’s psychosocial well-being. It has been frequently employed in psychological humor research around the world, as indicated by the various adaptations of the HSQ, for example in Arabic, Armenian, Chinese, French, Italian, Lebanese, and Turkish (Bilge & Saltuk, 2007; Chen & Martin, 2007; Kazarian & Martin, 2006; Saroglou & Scariot, 2002; Sirigatti, Penzo, Giannetti, & Stefanile, 2014; Taher, Kazarian, & Martin, 2008).

The present study investigates the psychometric properties (internal consistency and factor structure) of the German adaptation of the HSQ by combining five samples from Germany and Switzerland. The German HSQ has already been used in several published and unpublished studies (e.g., Martin et al., 2003; Ruch, Beermann, & Proyer, 2009; Samson, Huber, & Ruch, 2013) and typically the psychometric criteria were examined and reported. However, no formal adaptation study has been published until now. This will be done by drawing several samples from the German-speaking countries together. In addition, separate analyses are conducted for gender, age, and country to test the psychometric properties of the HSQ in these specific populations, and to test the factorial invariance for the first time. The norms provided can be used as a comparison standard in future studies with the German HSQ.

The second contribution of the present paper is to outline the theoretical similarity of the HSQ humor styles with other conceptualizations of styles of humor and to test their empirical overlap. Although the HSQ is currently the most frequently employed approach to humor styles, Wolfgang Schmidt-Hidding already presented eight comic styles (whereby “comic” broadly referred to humor) in 1963, which he derived from literature analyses. In 1996, Craik, Lampert, and Nelson proposed 10 styles of everyday humorous conduct, which can be arranged on five bipolar dimensions. Although the three approaches refer to styles of humor, their origins and frameworks differ from each other. Comparing the four humor styles of the HSQ with the eight comic styles and the ten styles of everyday humorous conduct highlights the similarities and differences of humor concepts using the label “styles”.

### Internal Consistency and Factorial Validity of the HSQ Scales

When developing the HSQ, Martin et al. (2003) employed a construct-based scale construction approach (see Jackson, 1970), which should lead to reliable and construct-valid measurement scales. They examined the literature on humor and well-being and derived four humor styles. The affiliative and self-enhancing humor styles are supposed to be benign to oneself and others, while the former should be used to improve one’s relationships with others and the latter to enhance oneself. The aggressive humor style is supposed to be detrimental to others and should enhance oneself, while the self-defeating humor style should be detrimental to oneself and improve one’s relationships with others.

Martin et al. (2003) refined the item pool to measure the four humor styles using several samples to arrive at the final 32-item solution, the HSQ. Combining these samples (*N* = 1,195), they obtained sufficient internal consistencies (Cronbach’s alpha ranging from .77 for aggressive to .81 for self-enhancing), which were replicated in other studies (e.g., Baughman et al., 2012). Occasionally, lower internal consistencies were obtained, especially in translated versions of the HSQ aggressive scale. Kazarian and Martin (2004), Sirigatti et al. (2014), and Chen and Martin (2007) found internal consistencies of .56, .58, and .61 for the HSQ aggressive scale, respectively, while the other three scales showed higher internal consistencies (≥ .70). Bilge and Saltuk (2007) reported acceptable internal consistencies for affiliative and self-enhancing (.74 and .78) and lower ones for aggressive (.69) and self-defeating (.67), and Taher et al. (2008) found acceptable internal consistencies for the self-enhancing and self-defeating scales (.89 and .76), and lower values for the affiliative (.67) and aggressive scales (.55). Based on these findings that internal consistencies were mostly high with the exception of the aggressive scale, the following prediction is made:

Prediction 1: The four HSQ scales show acceptable internal consistencies (Cronbach’s alpha ≥ .70). The lowest values are expected for the aggressive scale.

Factor analysis of the HSQ items should yield a four-factor simple structure, in which high loadings should be obtained for the items belonging to each scale, and the cross-loadings should be small. This four-factor structure has been supported in several studies employing principal components analyses (PCA; Bilge & Saltuk, 2007; Chen & Martin, 2007; Kazarian & Martin, 2004, 2006; Martin et al., 2003; Saroglou & Scariot, 2002; Taher et al., 2008) and confirmatory factor analyses (CFA; Chen & Martin, 2007; Martin et al., 2003; Sirigatti et al., 2014).

Translations into different languages often yield items that do not perform well, either due to an imprecise translation or due to cultural differences. In the HSQ research, occasionally a few items deviated from the expected simple structure in PCA; that is they had higher loadings on a non-corresponding humor style factor than on their corresponding factor. Saroglou and Scariot (2002) reported that one (unidentified) item deviated from simple structure. Kazarian and Martin (2004) detected two such items, Item 28 of the self-defeating scale (“If I am having problems or feeling unhappy, I often cover it up by joking around, so that even my closest friends don’t know how I really feel”) and Item 19 of the aggressive scale (“Sometimes I think of something that is so funny that I can’t stop myself from saying it, even if it is not appropriate for the situation”). Chen and Martin (2007) found deviations from simple structure for Item 28 of the self-defeating scale and Items 19 and 27 (“If I don’t like someone, I often use humor or teasing to put them down”) of the aggressive scale. Kazarian and Martin (2006) found mismatches for Item 9 of the affiliative scale (“I rarely make other people laugh by telling funny stories about myself”), Item 28 of the self-defeating scale, and Item 11 of the aggressive scale (“When telling jokes or saying funny things, I am usually not very concerned about how other people are taking it”). Taher et al. (2008) obtained mismatches for the following items: Item 28 (self-defeating), Item 9 (affiliative), and Items 3 (“If someone makes a mistake, I will often tease them about it”), 7 (“People are never offended or hurt by my sense of humor”), 11, and 27 (aggressive). Typically these items were already comparatively lower in the original study (Martin et al., 2003) despite displaying sufficient results there. In sum, 5 of the 32 items showed deviations from simple structure in two or more studies. Of the seven items listed at least once, five are from the aggressive scale (one each from the affiliative and self-defeating scales), corroborating the finding that the aggressive scale occasionally yielded a lower alpha. Based on these results, the following prediction is made:

Prediction 2: The factorial validity of the HSQ is supported a PCA and CFA, which are computed in separate samples (i.e., random halves of the total sample). Deviations from simple structure might be obtained for up to five items.

### Psychometric Properties in Demographic Groups

In general, it is important to determine whether a scale shows differences in demographic variables to avoid biases in results due to the sample composition and to allow for meaningful comparisons across different groups. First, we test if sufficient internal consistencies can be obtained in different demographic groups, specifically gender (males and females), three age groups (17–24, 25–35, and 36+ years), and country (Germany and Switzerland). Sirigatti et al. (2014) found that males and females as well as two age groups (adolescents and young adults) had good internal consistencies in all humor styles (.70–.85), except for the aggressive scale (.50–.61), with the lowest values obtained for females. In terms of differences between Germany and Switzerland, the above reviewed findings of the internal consistencies in different languages and cultures suggest values ≥ .70, with the possible exception of the HSQ aggressive scale. Based on these results, the following prediction is made in terms of the internal consistencies in the demographic groups:

Prediction 3: The four HSQ scales show acceptable internal consistencies for the demographic groups, gender, the three age groups, and the two countries (Cronbach’s alpha ≥ .70). We expect the lowest values for the aggressive scale.

Second, the means of the four HSQ scales might differ in the demographic groups. The results by Martin et al. (2003) indicate that means were significantly higher for males than for females. Effect sizes were small for affiliative, self-enhancing, and self-defeating (Cohen’s *d* < 0.20) and medium to large for aggressive (Cohen’s *d* = 0.72). Baughman et al. (2012) replicated these gender differences. Others found significant gender differences (males obtaining higher values than females) for specific subscales only, namely for the aggressive scale (Cohen’s *d* = 0.39, Bilge & Saltuk, 2007; Cohen’s *d* = 0.43, Sirigatti et al., 2014) or the aggressive and self-defeating scales (Cohen’s *d* = 0.31 and 0.21, Kazarian & Martin, 2004). Some found no gender differences (Chen & Martin, 2007) or that females scored higher than males in the affiliative scale (Cohen’s *d* = 0.20; Bilge & Saltuk, 2007). Although the findings on gender differences in the HSQ scales were in general mixed, the most consistent effect was that males scored higher in the aggressive scale than females.

Prediction 4: Males score higher in the HSQ aggressive scale than females.

In terms of age differences, Martin et al. (2003) compared participants younger than 19 years with those older than 25 years. They found that younger participants scored significantly higher in the affiliative (Cohen’s *d* = 0.72) and aggressive scale (Cohen’s *d* = 0.85). Older women scored higher than younger women in the self-enhancing scale (Cohen’s *d* = 0.29). Chen and Martin (2007) obtained similar age differences in participants younger than 22 and older than 23 years: Younger participants had higher scores than older ones in the affiliative (Cohen’s *d* = 0.27) and aggressive scales (Cohen’s *d* = 0.35). Thus, the affiliative and aggressive scores were found to be higher in younger participants, rather than in older participants in the two studies. The present studies uses a wider age range and treats age as a continuous variable instead of separating it into age groups. The following prediction is postulated for age-related differences in the four humor styles:

Prediction 5: Age correlates negatively with the HSQ affiliative and aggressive scales.

A final comparison of mean differences in the HSQ scales relates to people from two German-speaking countries, Germany and the German-speaking area of Switzerland. As this has not been previously investigated, we postulate an exploratory research question:

Research Question 1: Are there mean differences in the HSQ scales between Switzerland and Germany?

Third, testing factorial invariance (Cheung & Rensvold, 1999; Vandenberg & Lance, 2000) across different groups can determine whether the same factor structure holds across the different demographic groups (weak factorial invariance or configural invariance) and whether the groups have similar factor loadings (strong factorial invariance or metric invariance) or not. Support for at least weak factorial variance is needed to allow comparisons across the different groups (Cheung & Rensvold, 1999) and would further support the factorial validity of the HSQ. Factorial invariance can be tested using multi-group CFA by comparing the fit indices of a model that imposes the same factor structure across the different groups (Model 1, weak factorial invariance) and a model that imposes the same factor loadings across the different groups (Model 2, strong factorial invariance).

Martin et al. (2003) compared the varimax-rotated factor solution of the PCA for a subset of males (*n* = 177) and females (*n* = 275). High congruence coefficients (> .97) indicated that the four-factor structure was comparable across gender, which might hint to the presence of weak factorial invariance. However, we are not aware of any studies with the HSQ that tested the factorial invariance of the four scales multi-group CFA across any demographic groups.

Research Question 2: Can weak or strong factorial invariance be supported across the demographic groups of gender (males and females), three age groups (17–24, 25–35, and 36+ years), and two countries (Germany and Switzerland)?

### HSQ Humor Styles and Other Styles of Humor

The second goal, next to testing the psychometric properties of the German adaptation of the HSQ, is to theoretically outline and empirically investigate the relation of the HSQ scales to the similarly labeled conceptualizations of styles of everyday humorous conduct (Craik et al., 1996) and literary comic styles (Schmidt-Hidding, 1963). The term “style” has been used differently in psychology and humor research, and we chose the usage of styles in the context of traits as a unifying framework. This was deemed suitable as the three humor-related styles used in the present study can all be construed as trait-like, that is, as rather stable across time within a person, and as showing individual differences between people. To understand similarities and differences among these humor-related styles, the term style as construed by each of these authors is delineated and compared to the understanding of styles in the context of traits.

#### Definition of Styles in the Context of Traits

The typical use of the term *traits* refers to *content traits*, which reflect behavioral tendencies, that is, to what extent certain behaviors are demonstrated. For example, an extraverted person tends to be outgoing, talkative, and to attend social events, while an introverted person would show these behaviors less often. By contrast, *styles* denote *how* behavior is performed, and *stylistic traits* refer to the typical and habitual ways in which a person performs behavior (Buss & Finn, 1987). For example, Buss and Finn (1987) described playfulness in their classification as a stylistic trait as follows (p. 439):

“Playful people waggle their eyebrows, wink, roll their eyes, smirk, laugh raucously, make facetious remarks, and may exaggerate their movements in caricature. At the other extreme, serious people maintain a firm jaw, a set mouth, straight eyebrows, and in general, avoid silliness and retain a sober mien.”

As can be seen from this description, stylistic traits go beyond which behaviors are shown (e.g., playful people laughing more than serious ones), but playful people are described as more expressive and performative in their laughter than serious ones. While this description elaborates on playfulness at a broad level, humor styles–conceptualized as stylistic traits–should differentiate multiple ways in which humorous behavior can be performed. For example, one could tell jokes expressively, deadpan, restrained, or offensively. Thus, in the trait definition of styles and stylistic traits, humor styles should denote the typical ways in which a person performs humorous behavior.

#### Humor Styles

Martin et al. (2003) describe their definitions of humor styles as “individual differences in uses of humor” (p. 48), “distinctive uses or styles of humor” (p. 50), “functions, forms, or styles of humor” (p. 51), and “ways in which people use humor“ (p. 70). The stylistic aspect is added by describing that these functions or uses of humor can be either achieved in a way that is self-accepting (affiliative), tolerant of others (self-enhancing), detrimental to others (aggressive) or detrimental to the self (self-defeating) (Martin et al., 2003, p. 52). For example, a person could make a joke in a self-accepting way that improves one’s relationships with others (affiliative), or in way that is tolerant of others and enhances the self (self-enhancing), or in a way that is detrimental to others and enhances the self (aggressive), or in way that is detrimental to the self and enhances one’s relationships with others (self-defeating).

The stylistic aspect in the HSQ humor styles is thus based on evaluations of humor to achieve certain means. Both the evaluations and the functions can be either conscious and explicit or unconscious and implicit. This view on styles deviates from the trait definition in emphasizing different ways in which people use humor for a certain means and in the evaluations of this use, instead of different ways to *perform* the humor behavior itself. The latter would be closer to observable behavior (like making a deadpan face when telling a joke), while the HSQ humor styles rather focus on how humor is judged (like being self-accepting when telling a joke), adding an evaluative and more abstract dimension to the humor styles. Also, the emphasis on functions is unique to the HSQ humor styles.

#### Ten Styles of Everyday Humorous Conduct

Craik et al. (1996) define their styles of everyday humorous conduct by specific qualities or forms of behaviors and characteristics associated with humor. This understanding of styles is similar to the stylistic trait definition. Their instrument, the Humorous Behavior Q-Sort Deck (HBQD), contains 100 statements that should collectively provide information about a person’s humor style. These statements contain ideas from humor literature, tendencies of humor behavior, as well as various aspects of humor appreciation, comprehension, and production.

A PCA of Q-sort ratings (forced normal distribution from *very uncharacteristic* to *very characteristic*) of the HBQD statements resulted in five factors reflecting bipolar styles of humorous conduct. These were labeled socially warm vs. cold (socially constructive uses of humor vs. remaining socially aloof), reflective vs. boorish (recognizing and appreciating humor vs. competitive humor production), competent vs. inept (being witty vs. not being witty), earthy vs. repressed (enjoying vs. rejecting humor with sick, off-color, or sexual contents), and benign vs. mean-spirited (appreciating intellectual and harmless humor vs. mocking, joking about, and laughing at others).

Thus, both the definitions and dimensionality (four unipolar vs. five bipolar factors) of the HSQ humor styles and the styles of humorous conduct differ. The HSQ focuses on how people evaluate humor behaviors and the uses that these behaviors fulfill, whereas the HBQD focuses more on which humor behaviors are shown and how they are performed.

One study (*N* = 167 German adults) empirically investigated the relationship between the HSQ humor styles and the styles of everyday humorous conduct (Ruch, Proyer, Esser, & Mitrache, 2011). Using the HBQD Rating Form (HBQD-RF), which employs a normative instead of an ipsative response format, they found positive correlations between the HSQ affiliative scale and the HBQD-RF socially warm vs. cold, competent vs. inept, and earthy vs. repressed scales (medium to large effects). The HSQ self-enhancing scale showed small to medium correlations with four of the five HBQD-RF scales (all except for benign vs. mean-spirited). The HSQ aggressive scale correlated positively with reflective vs. boorish and earthy vs. repressed, and negatively with benign vs. mean-spirited (small to medium effects). Finally, the HSQ self-defeating scale correlated positively with earthy vs. repressed and negatively with benign vs. mean-spirited (small effects).

The pattern of correlations in Ruch et al.’s (2011) study showed that (a) the HSQ affiliative and the HBQD-RF socially warm vs. cold scales overlapped to a large extent, while the other correlations were of small to medium size, (b) the HBQD-RF reflective vs. boorish scale was relatively independent of the HSQ scales, and (c) all HSQ scales went along with the HBQD-RF earthy vs. repressed scale, which is surprising as the HSQ items do not contain any such content (i.e., sick, off-color, or sexual humor). The present study aims at replicating these findings using the HBQD-RF with 10 unipolar scales instead of five bipolar ones. This was preferred as Ruch et al. (2011) found that some of the HBQD-RF scales were not strictly bipolar, and the ten unipolar scales should thus provide a better interpretation as to which end of the poles might be more or less overlapping with the HSQ humor styles; for example, the mean-spirited pole might be more relevant for HSQ aggressive scale than the benevolent one. The following predictions are derived from the previous findings:

Prediction 6: The four HSQ scales show similar correlations to the ten unipolar scales of the HBQD-RF as they did to the five bipolar ones in the Ruch et al. (2011) study. However, it is not expected that the correlations are symmetric for the styles of humorous conduct at both ends of the poles.

Research Question 3: How much variance do the four HSQ scales explain in each of the ten unipolar scales of the HBQD-RF?

Research Question 4: How much variance do the ten unipolar scales of the HBQD-RF explain in each of the four HSQ scales?

#### Eight Comic Styles

Another tradition of humor-related styles stems from literary studies, in which a comic style (or style of comedy) refers to “the nature or ‘flavor’ of comedy and humor in general” (Milner Davis, 2014, p. 264). The term comic (German: “Komik”) in this sense is similar to the umbrella definition of humor used in psychological research, in which all comical and humor-related phenomena are subsumed.

Schmidt-Hidding (1963) described the characteristics and conduct associated with eight comic styles as prevalent in literary studies, namely fun, humor (in the narrow sense of benevolent humor), nonsense, wit, irony, satire, sarcasm, and cynicism. He also presented a model distinguishing these eight styles according to seven characteristics, namely the (a) intention and goal associated with each comic style, (b) the object or topic of the style, (c) the attitude of the person showing the comic style, (d) the person’s behaviors towards other people, (e) the ideal audience, (f) the method or procedure of showing the comic style, and (g) linguistic peculiarities. Although transcending the definition of stylistic traits, these eight comic styles also entail how humorous behavior is performed (e.g., teasing, mocking, surprising, brief, ambiguous).

The list of eight comic styles is not exhaustive, yet it was taken as a starting point for assessing individual differences in the endorsement of comic styles (Ruch, 2012). To this end, Schmidt-Hidding’s descriptions were transformed into self-ratings for the present study, called the Comic Styles Rating Form. It consists of short texts describing each comic style, and participants provided ratings on how frequently they show each of them. Testing the overlap of comic styles with the HSQ is especially interesting, as they stem from different disciplines (psychology vs. linguistics) and were developed using different rationales (literature on humor and well-being with an empirical scale construction vs. a lexical and rational approach). As with the styles of everyday humorous conduct, the comic styles refer more to humor behaviors and how they are performed, which results in similar differences to the HSQ humor styles.

However, some similarities regarding the contents can also be delineated. The HSQ affiliative humor style and the comic style fun share an emphasis on social humor production, and the HSQ self-enhancing scale entails a component of recognizing absurdities in life, which is also a part of the comic style humor. Finally, the HSQ aggressive scale and the comic styles satire, sarcasm, and cynicism all refer to mocking, criticizing, and making fun of others. No equivalent to the HSQ self-defeating humor styles can be delineated in the comic styles. Conversely, it is assumed that the comic styles primarily related to linguistic peculiarities or cognitive factors, such as nonsense, wit, and irony, will have no direct correspondence in the HSQ. Based on the conceptual considerations, the following prediction and research questions are postulated:

Prediction 7: The HSQ affiliative humor style correlates positively with fun, self-enhancing with humor, and aggressive with satire, sarcasm, and cynicism.

Research Question 5: How much variance do the four HSQ scales explain in each of the eight comic styles?

Research Question 6: How much variance do the eight comic styles explain in each of the four HSQ scales?

## Method

### Sample

Five samples (*n* = 75–382, entailing published data from Ruch & Heintz [2013] and unpublished data) were recruited by different researchers from Swiss and German universities, employing the German adaptation of the HSQ. They were combined into a total sample of *N* = 1,101 (27.3% males), ranging in age from 16 to 76 (*Mdn* = 25, *M* = 29.37, *SD* = 11.36). Participants were primarily from Switzerland (61.2%) and Germany (23.6%), while 15.2% were from other countries or did not indicate a country. Participants were overall well educated, with 37.1% having more than 13 years of education, 59.9% having 10–13 years of education, and 27% having up to 9 years of education (1.3% did not provide sufficient information about their education). A subsample of 344 participants (36.6% males) provided ratings on the HBQD-RF and 285 participants (35.8% males) provided ratings on the Comic Styles Rating Form.

### Instruments

#### HSQ

The HSQ (Martin et al., 2003) consists of 32 items measuring four humor styles (8 items each). The Likert-type response scale ranges from *totally disagree* (1) to *totally agree* (7). The German adaptation of the HSQ was done in several steps, which were monitored and guided by an expert in humor and assessment: (a) A team of several students (German native speakers, proficient English skills) independently translated the original English version of the HSQ into German. (b) Another team of students (including a linguist) provided independent back translations. (c) Differences between the original English version and the back-translations were discussed and resolved across the student teams and the expert. (d) This version was empirically assessed and items were further refined (e.g., clarity, understandability) to provide the final German adaptation of the HSQ. This version can be obtained by contacting the authors.

#### HBQD Rating Form

The HBQD Rating Form (HBQD-RF, Ruch et al., 2009) contains the 100 statements of the HBQD (Craik et al., 1996) measuring ten unipolar styles of humorous conduct with 6–15 items each: Socially warm, socially cold, reflective, boorish, competent, inept, earthy, repressed, benign, and mean-spirited. The bipolar Likert-type response scale ranges from *very uncharacteristic* (1) to *very characteristic* (7). The intercorrelations between the ten scales ranged from -.38 (socially warm and social cold) to .70 (earthy and mean-spirited), with a median of .29.

#### Comic Styles Rating Form

The Comic Styles Rating Form consists of eight short texts containing the characteristics of the comic styles as described by Schmidt-Hidding (1963): Fun, humor, nonsense, wit, irony, satire, sarcasm, and cynicism. An example text (for nonsense) is: “When using nonsense humor, I’d like to demonstrate how ridiculous the bare mind is. But basically, nonsense is absolutely pointless. When employing nonsense, my relation to language can be described as inventive.” Participants rated how frequently they show these characteristics on a Likert-type scale from *never* (1) to *very often* (5). The intercorrelations between the eight ratings ranged from -.18 (irony and humor) to .58 (sarcasm and cynicism), with a median of .05.

### Procedure

The questionnaires were completed online or with paper-pencil format between 2009 and 2012. The HSQ was usually one questionnaire within a larger study.

### Analyses

The internal consistencies of the total sample (Prediction 1) and the subgroups (Prediction 3) are indicated by Cronbach’s alpha. The factorial validity of the HSQ (Prediction 2) is tested with PCA and CFA (each conducted in one random half of the total sample), the latter modeled with the *lavaan* package (Rosseel, 2012) in *R* (R Development Core Team, 2015). The robust MLM estimator (with Satorro-Bentler corrections) was employed, and the following fit indices are reported (with the values in parentheses indicating a good and acceptable fit according to Schermelleh-Engel, Moosbrugger, & Müller, 2003): χ^2^ (good: *p* > .05, acceptable: *p* ≥ .01), χ^2^/*df* (good: ≤ 2, acceptable: ≤ 3), Comparative Fit Index (CFI; good: ≥ .97, acceptable: ≥ .95), Root Mean Square Error of Approximation (RMSEA; good: ≤ .05, acceptable: ≤ .08), and Standardized Root Mean Square Residual (SRMR; good: ≤ .05, acceptable: ≤ .10). Weak or strong factorial invariance (Research Question 2) is tested in a multi-group CFA. The two models of factorial invariance are computed by introducing the relevant group factors into the model, forcing the same factor structure across groups (weak factorial invariance), and by additionally forcing all loadings to be equal across groups (strong factorial invariance). These models are then compared using the χ^2^ difference test (using scaled statistics according to Satorra & Bentler, 2010) and the Akaike Information Criterion (AIC) values. Gender (Prediction 4) and country differences (Research Question 1) in the means of the HSQ scales are investigated with unpaired *t*-tests. The correlations of the HSQ scales with age (Prediction 5), the HBQD-RF scales (Prediction 6), and the comic styles (Prediction 7) are analyzed with Pearson correlation coefficients. The variance that the four HSQ scales explain in the HBQD-RF scales (Research Question 3) and the comic styles (Research Question 5) and vice versa (Research Questions 4 and 6) is analyzed with standard multiple regression analyses.

## Results

As the five samples were recruited by different researchers, we first tested whether they all measure the HSQ scales reliably and show an appropriate factor structure to avoid biases. Scale analyses showed that the internal consistencies were comparable across the samples (ranging from .79–.91 for affiliative, .80–.87 for self-enhancing, .69–.74 for aggressive, and .77–.87 for self-defeating), and all corrected item-total correlations were positive. PCA with varimax rotation in each sample supported the four-factor structure of the HSQ, with only 1 to 4 loadings per sample (i.e., 3.1–12.5% of the items) deviating from simple structure (i.e., higher loadings on another but the intended HSQ factor). Given these comparable and sufficient psychometric properties within each sample, we combined them into the total sample of 1,101 participants. Note that this total sample was split in two random halves for the analyses of the PCA (*n* = 550) and CFA (*n* = 551).

### Internal Consistency and Factorial Validity of the HSQ

Predictions 1 and 2 refer to the internal consistency and factorial validity of the German adaptation of the HSQ in the total sample. Table 1 shows the internal consistencies (Cronbach’s alpha) of the four HSQ scales.

**Table 1:** Internal Consistencies (Cronbach’s Alpha) of the Four Scales of the German Humor Styles Questionnaire (HSQ) in the Total Sample and in Several Demographic Subgroups

HSQ scale	Total	Gender		Age group			Country	
		M	F	16–24	25–35	36+	CH	GER
*N*	1,101	301	800	517	343	241	674	260
Affiliative	.87	.89	.86	.84	.89	.87	.86	.87
Self-enhancing	.83	.80	.84	.82	.83	.83	.81	.85
Aggressive	.70	.73	.67	.72	.66	.68	.70	.72
Self-defeating	.81	.80	.81	.82	.80	.80	.81	.82

*Note.* M = males; F = females; CH = Switzerland; GER = Germany.

As can be seen in Table 1, the four HSQ scales had good internal consistencies (Cronbach’s alpha ≥ .80), while the internal consistency of the aggressive scale was acceptable (.70). This confirms Prediction 1. The factorial validity of the HSQ in the total sample is tested with a PCA and CFA. As expected, the scree test and the parallel analysis suggested the retention of four factors (first eight eigenvalues: 5.89, 4.05, 2.80, 1.82, 1.32, 1.25, 1.08, 1.00), together explaining 45.5% of variance. The rotated component matrix and communalities of the PCA with varimax rotation is shown in Table 2.

**Table 2:** Rotated Component Matrix and Communalities of a Principal Components Analysis With Varimax Rotation and Standardized Loadings of a Confirmatory Factor Analysis of the 32 Items of the Four Scales of the German Humor Styles Questionnaire

	Principal components analysis					Confirmatory factor analysis			
HSQ item	F1 AF	F3 SE	F4 AG	F2 SD	*h*^2^	F1 AF	F2 SE	F3 AG	F4 SD
AF 1 (rec.)	**.77**	.12	.10	-.05	.62	**.69**			
AF 5	**.67**	.28	.07	.06	.53	**.73**			
AF 9 (rec.)	**.58**	.06	.01	.21	.39	**.45**			
AF 13	**.76**	.15	.00	-.05	.60	**.77**			
AF 17 (rec.)	**.66**	.08	.11	.00	.45	**.72**			
AF 21	**.75**	.15	.02	.10	.60	**.73**			
AF 25 (rec.)	**.73**	.08	.02	.02	.55	**.65**			
AF 29 (rec.)	**.67**	.24	.09	-.08	.51	**.77**			
SE 2	.26	**.59**	-.01	-.02	.42		**.74**		
SE 6	.26	**.52**	-.06	.07	.34		**.55**		
SE 10	.03	**.78**	-.02	.08	.61		**.79**		
SE 14	.28	**.60**	-.09	.01	.45		**.60**		
SE 18	.00	**.78**	-.08	.03	.62		**.78**		
SE 22 (rec.)	.19	**.51**	.07	-.09	.31		**.51**		
SE 26	.16	**.69**	-.02	.09	.51		**.66**		
SE 30	.03	**.52**	-.07	-.07	.28		**.46**		
AG 3	.08	.06	**.59**	.24	.41			**.60**	
AG 7 (rec.)	-.07	-.13	**.50**	.11	.28			**.40**	
AG 11	-.06	.18	**.19**	.03	.07			**.17**	
AG 15 (rec.)	.12	.00	**.72**	.07	.54			**.65**	
AG 19	.19	.20	**.18**	.23	.16			**.29**	
AG 23 (rec.)	.28	-.13	**.62**	-.01	.48			**.70**	
AG 27	-.05	-.03	**.60**	.16	.39			**.53**	
AG 31 (rec.)	.12	-.04	**.72**	.02	.53			**.65**	
SD 4	-.06	.00	.08	**.71**	.51				**.66**
SD 8	.11	-.02	.08	**.81**	.68				**.77**
SD 12	.10	.07	.12	**.66**	.47				**.62**
SD 16 (rec.)	.32	-.09	.15	**.54**	.42				**.48**
SD 20	-.13	-.01	.04	**.79**	.64				**.74**
SD 24	-.15	-.04	.06	**.62**	.41				**.56**
SD 28	-.21	.22	.12	**.31**	.20				**.32**
SD 32	.23	.05	.13	**.71**	.57				**.69**
% expl. var.	14.5	11.2	8.1	11.7					

*Note. n* = 550 (PCA) and *n* = 551 (CFA). AF = affiliative; SE = self-enhancing; AG = aggressive; SD = self-defeating; rec. = recoded; *h*^2^ = communalities; % expl. var. = percent of explained variance by the factor (after rotation). Loadings of items theoretically belonging to one factor in bold.

As can be seen in Table 2, the loadings on each of the four factors was high for affiliative (.58–.77), self-enhancing (.51–.78), and self-defeating (.54–.81), with the exception of one item (Item 28 with a loading of .31). The aggressive scale had two items with low loadings (.18 for Item 19 and .19 for Item 11), while the other loadings were high (.50–.72). Second loadings were low (*Mdn* = .08), with only one item exceeding .30 (Item 16 from the self-defeating scale). Only Item 19 (aggressive scale) had higher second loadings than the loading on the aggressive factor (i.e., a deviation from simple structure). In general, communalities were high (*Mdn* = .48), exceeding .25 for all items except for the three that had low loadings on their intended factor (i.e., Items 11, 19, and 28). Thus, with one exception (Item 19), the four-factor structure of the HSQ was supported in the PCA. This was also confirmed by the very high congruence coefficients (Tucker’s Phi), comparing the four factors of the present PCA with those of the original sample (see Martin et al., 2003, pp. 58–59), which ranged from .94 (aggressive) to .98 (self-defeating).

A CFA was conducted with the other half of the sample (*N* = 551), resulting in a mostly acceptable model fit, χ^2^(458) = 1271.97, *p* < .001, χ^2^/*df* = 2.78, CFI = .841, RMSEA = .057, and SRMR = .076. Table 2 also shows the standardized factor loadings of the CFA. The results were similar to the PCA, with the same three items loaded < .40 on their corresponding factors (i.e., Items 11, 19, and 28).

In addition, the correlations between the four HSQ scales and the latent factors in the CFA are of interest, as they should not correlate too strongly with one another. In the PCA sample, the observed correlations were significant between affiliative and self-enhancing (*r* = .39, *p* < .001), affiliative and aggressive (*r* = .23, *p* < .001), affiliative and self-defeating (*r* = .10, *p* = .014), and between aggressive and self-defeating (*r* = .31, *p* < .001). In the CFA, three significant correlations emerged between affiliative and self-enhancing (*r* = .49, *p* < .001), affiliative and aggressive (*r* = .29, *p* < .001), and aggressive and self-defeating (*r* = .24, *p* < .001). Although medium to large correlations emerged between some of the humor styles (especially between affiliative and self-enhancing), the scales can still be regarded as distinguishable, yet not independent, humor styles. Overall, the factorial validity of the HSQ can be mostly supported in both the PCA and the CFA, confirming Prediction 2.

### Differences in Demographic Variables

Firstly, the reliabilities in the demographic subgroups gender, age, and country are investigated. As can be seen in Table 1, the internal consistencies of the HSQ scales in all subgroups exceed .80. As expected, the aggressive scale had lower values, which ranged from .66 to .73. Internal consistency was below .70 for females and for the age groups 25–35 and 36+ years. This confirms Prediction 3.

Next, the mean differences across gender, age, and countries are of interest. Table 3 shows the means and standard deviations of the four HSQ scales in the total sample and in the demographic subgroups.

**Table 3:** Means (With Standard Deviations in Parentheses) of the Four Scales of the German Humor Styles Questionnaire (HSQ) in the Total Sample and in Several Demographic Subgroups

Scale	Total	Gender		Country	
		Male	Female	CH	GER
*N*	1,101	301	800	674	260
AF	44.49 (8.07)	44.65 (8.70)	44.43 (7.83)	44.49 (7.71)	44.35 (8.60)
SE	36.04 (8.48)	36.11 (8.09)	36.01 (8.62)	36.42 (8.18)	35.45 (9.03)
AG	28.01 (7.58)	30.46_a_ (8.07)	27.10_b_ (7.17)	27.89 (7.57)	28.03 (7.74)
SD	25.39 (8.41)	26.02 (8.23)	25.15 (8.47)	25.25 (8.33)	25.54 (8.58)

*Note.* AF = affiliative; SE = self-enhancing; AG = aggressive; SD = self-defeating; CH = Switzerland; GER = Germany. Means with different subscripts in the subgroups differed significantly from one another (*p* < .05).

As can be seen in Table 3, males scored significantly higher in the HSQ aggressive scale (*p* < .001, Cohen’s *d* = 0.44), while the other three scales showed no gender differences (all *p*s > .12), confirming Prediction 4. No significant differences emerged between the participants from Switzerland and Germany (all *p*s > .13), answering Research Question 1. Age correlated negatively with the affiliative (*r* = -.11, *p* < .001), aggressive (*r* = -.19, *p* < .001), and self-defeating (*r* = -.08, *p* = .005) scales and positively with the self-enhancing scale (*r* = .09, *p* = .004). This only partly confirms Prediction 5, as every HSQ scale showed small age effects, not only the affiliative and aggressive ones.

Third, the factorial invariance of the HSQ is tested across gender, age, and country. Table 4 shows the fit indices of the models for weak and strong invariance and the χ^2^ difference test.

**Table 4:** Fit Indices of Models Assessing Weak (Model 1) and Strong (Model 2) Factorial Invariance of the German Humor Styles Questionnaire Across Several Demographic Subgroups

Subgroup, Model	χ^2^	χ^2^/*df*	CFI	RMSEA	SRMR	AIC	χ^2^ diff.
Gender (males and females)							50.30**
Model 1	2,607.67***	2.85	.83	.06	.07	120,998	
Model 2	2,663.43***	2.82	.83	.06	.08	120,995	
Age group (17–24, 25–35, 36+)							93.53**
Model 1	3,106.98***	2.26	.83	.06	.08	120,806	
Model 2	3,198.33***	2.24	.82	.06	.08	120,791	
Country (Switzerland and Germany)							33.80
Model 1	2,376.35***	2.59	.83	.06	.08	102,730	
Model 2	2,413.10***	2.56	.83	.06	.08	102,708	

*Note.* CFI = Comparative Fit Index; RMSEA = Root Mean Square Error of Approximation; SRMR = Standardized Root Mean Square Residual; AIC = Akaike Information Criterion; χ^2^ diff. = χ^2^ difference test.

**p < .01. ***p < .001.

As can be seen in Table 4, the models testing weak and strong factorial invariance showed an acceptable fit for all demographic groups, with the exception of the significant χ^2^ (*p* < .001) and the CFI (.82–.83) that indicated an unacceptable fit. The χ^2^ difference test was significant for gender and age, indicating that the model of strong factorial invariance fitted the data significantly worse than the weak invariance model. This supports weak factorial invariance across gender and the three age groups. By contrast, evidence was obtained for strong factorial invariance between the two countries, answering Research Question 2.

### HSQ Humor Styles and Other Styles of Humor

First, the four HSQ scales were correlated with the five bipolar styles of humorous conduct of the HBQD-RF. The results are shown in Table 5.

**Table 5:** Correlations of the Four Scales of the German Humor Styles Questionnaire (HSQ) With the Ten Unipolar Scales of the Humor Behavior Q-Sort Deck Rating Form (HBQD-RF) and Total Shared and Explained Variance in Standard Multiple Regression Analyses With Cronbach’s Alpha for (Comparison)

HBQD-RF scales	HSQ AF	HSQ SE	HSQ AG	HSQ SD	Total *R*^2^	*R*	α
Socially warm	.65***	.48***	.13*	.18***	.50***	.70***	.81
Socially cold	-.54***	-.31***	.18***	.19***	.39***	.62***	.74
Reflective	.34***	.32***	.17**	.20***	.20***	.44***	.73
Boorish	.28***	.15**	.31***	.35***	.22***	.47***	.72
Earthy	.10	-.02	.57***	.36***	.36***	.60***	.82
Repressed	-.21***	-.09	-.13*	.09	.08***	.27***	.29
Competent	.49***	.19***	.33***	.22***	.33***	.57***	.66
Inept	-.19***	-.11*	.20***	.33***	.17***	.41***	.74
Benign	.31***	.27***	.08	.07	.12***	.35***	.58
Mean-spirited	.08	-.03	.57***	.45***	.41***	.64***	.81
Total *R*^2^	.57***	.30***	.40***	.25***			
*R*	.76***	.55***	.63***	.50***			
Cronbach’s α	.83	.79	.69	.78			

*Note. N* = 344. AF = affiliative; SE = self-enhancing; AG = aggressive; SD = self-defeating. Total *R*^2^ = shared variance; *R* = explained variance.

*p < .05. **p < .01. ***p < .001.

As can be seen in Table 5, the correlations were mostly as predicted. The HSQ affiliative humor style mainly overlapped with the socially warm, socially cold (negative), competent (large effects) and inept (negative) styles. It correlated negatively with repressed (but not earthy) and also correlated positively with the benign style of humorous conduct (medium effects). Surprisingly, it correlated positively with both the reflective and boorish styles of humorous conduct (accounting for the zero correlation in the study by Ruch et al., 2011). The HSQ self-enhancing humor style was characterized by a reflective and benign style of humorous conduct (medium effects) in addition to the socially warm (large effect) and socially cold (negative) styles of humorous conduct. The HSQ aggressive humor style strongly overlapped with the earthy and mean-spirited styles of humorous conduct as well as with boorish and competent (medium effects). The HSQ self-defeating humor style showed the strongest correlations with mean-spirited, earthy, boorish, and inept (medium to large effects). This partly confirms Prediction 6, as the correlations obtained were lower for earthy and repressed and higher for reflective and boorish. The asymmetric correlations between the two poles of each of the bipolar styles of humorous conduct (and the positive correlations of both styles that is evened out in a bipolar dimension supports the importance of separating them into ten unipolar styles.

Examining the variance that was shared by the four HSQ scales in the ten unipolar styles of humorous conduct (Research Question 3), large effects were found in all styles of humorous conduct but the repressed and benign ones (medium to large effects). On average, the HSQ scales and the styles of humorous conduct shared 27.5% of the variance, with a maximum of 50.0% (socially warm style of humorous conduct). In terms of explained variance, the four HSQ humor styles explained between 27.0% (repressed) to 70.0% (socially warm) of the absolute variance in the styles of humorous conduct (*Mdn* = 52.0%), and between 55.4% (inept) and 93.1% (repressed) of their reliable variance (*Mdn* = 76.1%). In other words, on average one fourth of the variance was unique to the styles of humorous conduct and could not be explained by the four HSQ scales.

Large effects were also found when the ten styles of humorous conduct predicted the four HSQ scales (Research Question 4), with the two approaches sharing between 25.0% (self-defeating) to 57.0% (affiliative) of the variance (*Mdn* = 35.0%). The absolute variance predicted by the styles of humorous conduct was on average 59.0%, and the reliable variance explained ranged from 64.1% (self-defeating) to 91.3% (aggressive) and 91.6% (affiliative), with a median of 80.5%. Thus, on average around one fifth of the variance was unique to the HSQ humor styles and could not be explained by the ten styles of humorous conduct. These findings underscore that the HSQ and the HBQD-RF assess similar, but not exchangeable, humor-related styles.

Prediction 7 and Research Questions 5 and 6 concern the overlap of the four HSQ scales with the self-ratings of the eight comic styles. Table 6 shows the correlations of the HSQ with the eight scales of the Comic Styles Rating Form.

**Table 6:** Correlations of the Four Scales of the German Humor Styles Questionnaire (HSQ) With the Eight Scales of the Comic Styles Rating Form and Total Shared and Explained Variance in Standard Multiple Regression Analyses With Cronbach’s Alpha (for Comparison)

Comic styles	HSQ AF	HSQ SE	HSQ AG	HSQ SD	Total *R*^2^	*R*
Fun	.35***	.19**	.03	.10	.14***	.38***
Humor	.08	.24***	-.27***	.07	.18***	.42***
Nonsense	.13*	.12*	.07	.03	.03	.16
Wit	.07	.09	.35***	.13*	.13***	.36***
Irony	.01	-.09	.25***	.10	.08***	.28***
Satire	.03	-.06	.39***	.11	.16***	.41***
Sarcasm	-.01	-.05	.42***	.16**	.19***	.44***
Cynicism	-.09	-.13*	.39***	.19**	.20***	.44***
Total *R*^2^	.14***	.12***	.34***	.07**		
*R*	.38***	.34***	.58***	.27***		
Cronbach’s α	.80	.80	.69	.78		

*Note. N* = 285. AF = affiliative; SE = self-enhancing; AG = aggressive; SD = self-defeating. Total *R*^2^ = shared variance; *R* = explained variance.

*p < .05. **p < .01. ***p < .001.

As can be seen in Table 6, the HSQ affiliative humor style correlated positively with fun (medium effect) and nonsense (small effect). The HSQ self-enhancing style correlated positively with humor (medium effect), fun, nonsense and lower cynicism (small effects). The HSQ aggressive humor style overlapped with sarcasm, satire, cynicism, and wit (medium to large effects) and to a lesser extent with irony and low humor (small to medium effects). Lastly, the HSQ self-defeating scale showed small to medium positive correlations with cynicism, sarcasm, and wit. Thus, overall the stronger alignments of affiliative with fun and of aggressive with satire, sarcasm, and cynicism as postulated by Prediction 7 were substantiated. Also, the low correlations of the HSQ humor styles with nonsense and irony showed that these comic styles were rather independent of the HSQ, while medium to strong correlations were obtained for wit and satire with the HSQ aggressive humor style.

Examining the variance that is shared by the four HSQ scales in the eight comic styles (Research Question 5), medium to large effects were found for all comic styles but nonsense (small effect). On average, the HSQ scales and the styles of humorous conduct shared 15.0% of the variance, with a maximum of 20.0% (cynicism). In terms of explained variance, the four HSQ humor styles explained between 16.0% (nonsense) to 44.0% (sarcasm and cynicism) of the absolute variance in the styles of humorous conduct (*Mdn* = 39.5%).

Medium to large effects were also found when the eight comic styles predicted the four HSQ scales (Research Question 6). The two approaches shared between 7.0% (self-defeating) and 34.0% (aggressive) of the variance (*Mdn* = 13.0%). The absolute variance predicted by the styles of humorous conduct was on average 36.0%, and the reliable variance explained ranged from 34.6% (self-defeating) to 84.1% (aggressive), with a median of 45.0%. Thus, on average around half of the variance was unique to the HSQ humor styles and could not be explained by the eight comic styles. The lower coefficients are, of course, partly due to the fact that the comic styles were measured with one item only, which lowers reliability. Again, the HSQ humor styles and comic styles were not exchangeable.

## Discussion and Conclusion

The present study aimed at testing the psychometric properties of the German adaptation of the HSQ. The second goal was to examine the theoretical and empirical overlaps with two previous conceptualizations of styles in humor research, namely styles of humorous conduct (Craik et al., 1996) and comic styles (Schmidt-Hidding, 1963).

The internal consistencies obtained were good for all HSQ scales and satisfying for the aggressive scale, supporting Prediction 1. The factorial validity was supported in a PCA (including the high congruence coefficients) and CFA, with three items showing low loadings (Items 11, 19, and 28) and one item violating simple structure in PCA (Item 19 of the aggressive scale). These items showed similar deviations from simple structure or low loadings in previous studies (Chen & Martin, 2007; Kazarian & Martin, 2004, 2006; Saroglou & Scariot, 2002; Taher et al., 2008). Thus, a more general difficulty of fitting these items into the four-factor structure is a more likely explanation than specific problems with the German translation or cultural differences. Compared to the other items of the respective scales, these three items were also the lowest two in the aggressive scale, and the lowest in the self-defeating humor style scales in the PCA by Martin et al. (2003). This also sheds light on the reasons for the aggressive scale consistently yielding a comparably lower internal consistency. Overall, however, the factor structure of the HSQ was replicated in the PCA and CFA, supporting Prediction 2.

The item content can potentially help to explain why the three items did not fit as expected into the four-factor structure in the present and previous studies. Item 11 (“When telling jokes or saying funny things, I am usually not very concerned about how other people are taking it.”) of the aggressive scale does not directly involve disparaging humor, as not being concerned how others take it could also mean–amongst other things–that one makes jokes that others don’t find funny. Thus, people can interpret this item in an aggressive or in less aggressive way. Item 19 (“Sometimes I think of something that is so funny that I can’t stop myself from saying it, even if it is not appropriate for the situation.”) of the aggressive scale does also not directly involve laughing at or mocking others, but rather being impulsive and saying something funny that is inappropriate. Thus, this item is lacking a directly aggressive connotation, which could explain why this item occasionally loads higher on other humor styles than the aggressive one. Item 28 (“If I am having problems or feeling unhappy, I often cover it up by joking around, so that even my closest friends don’t know how I really feel.”) of the self-defeating scale might yield lower loadings, as it is the only item of this scale that entails the aspect of hiding one’s negative feelings. This aspect seems to be less related to the other self-defeating items in translated versions than in the original study.

Thus, the content of these three items deviates from the other scale items, which could explain why these items did not load as highly on their corresponding humor style factors and why the aggressive scale also shows lower internal consistencies in several studies. Also it is noteworthy that the three items stemmed from the supposedly “maladaptive” HSQ scales (aggressive and self-defeating). An alternative interpretation as to why these three items deviate in both the current and previous samples is that they are rather long and contain conditional clauses^i^, which might introduce additional complexities in interpreting these items and in translating them appropriately.

### Influence of Demographic Groups

Next, the psychometric properties of the German HSQ across several demographic groups were of interest. The internal consistencies were good except for the aggressive scale, which was lower than .70 for three demographic groups. Obtaining lower values for females than for males replicated the findings with the Italian version of the HSQ (Sirigatti et al., 2014). The mean differences in the HSQ scales were significant for the aggressive scale only, with males obtaining higher values than females, confirming Prediction 4. Age differences were small but in the expected directions, with affiliative, and aggressive decreasing with age. In addition, self-enhancing increased and self-defeating decreased with age. The latter two humor styles however shared less than 1% of the variance with age, thus–despite their statistical significance (likely due to the large sample size)–they were not practically meaningful. This partially confirms Prediction 5 and extends previous findings on age differences in the HSQ, which typically compared only two age groups (adolescents and younger/middle-aged adults) with one another (Martin et al., 2003; Sirigatti et al., 2014). That is, the means of the HSQ varied even if different groups of adults were compared with one another (from emerging adults to older adults). The exploratory comparison of people from Switzerland and Germany (Research Question 1) revealed no significant mean differences.

The present study also investigated the factorial invariance of the HSQ across three demographic groups (Research Question 2). Strong factorial invariance was found across the two countries (Switzerland and Germany); that is, participants from both countries had the same four-factor structure and the same factor loadings. Weak factorial invariance was supported across gender and the three age groups. That is, they shared the same factor structure, but the loadings were different across the groups. Thus, differences across these demographic groups can be meaningfully compared in relation to the underlying four humor style factors, as they have “the same conceptual frame of reference” (Vandenberg & Lance, 2000, p. 37).

This finding is important as other differences obtained between the groups (e.g., between males and females) could also be due to a different composition of the four HSQ factors, in which for example the aggressive scale would be composed of different items for each group. This would make it challenging to differentiate between “true” differences in the HSQ factors and simply different compositions of these factors. However, our findings show that–at least for the German adaptation of the HSQ–the same factor structure holds for gender, age groups, and nationalities (the latter also having the same loadings). For the groups in which strong factorial invariance was not given, future studies could test for partial metric (or partial strong factorial) invariance to determine for which HSQ items the same loadings hold across the demographic groups.

Overall, the German adaptation of the HSQ can be considered successful. Internal consistencies were satisfying in the overall sample and for the demographic subgroups. Apart from one item of the HSQ aggressive scale, the four-factor structure was supported and at least weak factorial invariance was given across gender, age groups (16–24, 25–35, and 36+), and countries (Germany and Switzerland). The adaptation can thus be recommended for further studies with the HSQ in German-speaking countries. Although the sample of Austrians was not large enough to allow for separate multi-group analyses, we expect–based on the encouraging findings across Switzerland and Germany–that the psychometric properties will also be sufficient in Austria. This prediction could be empirically tested in future studies.

### HSQ and Other Styles of Humor

In addition to investigating the psychometric properties of the German version of the HSQ, the overlaps between the HSQ humor styles and two previous conceptualizations of styles of humor were of interest. In general, two of the HSQ humor styles showed stronger overlaps with the previous concepts of styles of humor: The aggressive scale had its counterparts in both the styles of everyday humorous conduct (earthy und mean-spirited) and in the comic styles (wit, satire, sarcasm, and cynicism) and the affiliative scale corresponded with several styles of humorous conduct (e.g., socially warm, socially cold, and competent) and the comic style fun. As expected, self-enhancing related to the comic style (benevolent) humor, and in the HBQD-RF, and it was an amalgam of socially warm, reflective and benign humorous conduct. There was no direct counterpart for the self-defeating humor style; but spurious relations were present to boorish, earthy, inept and even mean-spirited styles of everyday humorous conduct. Although it is not a comic style in literature studies, it tended to be endorsed by those high in the comic styles sarcasm and cynicism. This style of humor seemed more unique to the HSQ and deserves more attention in future studies.

Furthermore, correlations with the ten unipolar scales of the HBQD-RF were of larger magnitude than expected, although the pattern largely conformed to the previous findings obtained with the bipolar styles of humorous conduct (Ruch et al., 2011). The importance of separating the bipolar styles into unipolar ones was highlighted by the fact that most correlations were asymmetric across the poles (e.g., the HSQ affiliative scale correlated .49 with competent and .19 with inept). This might also be the reason why the obtained correlations were in general larger than expected. In particular, if the bipolar styles of humorous conduct are not perfectly bipolar (as found by Ruch et al., 2011, and as evidenced by the largest negative correlation being -.38 in the present study), and if the HSQ humor styles overlap more with one end of the pole or correlate positively with both poles, then using the bipolar styles of humorous conduct lowers the correlations that can be obtained or it might mask existing correlations (as we found for the reflective and boorish styles of humorous conduct).

The present findings might thus be a more realistic estimate of the overlaps between the HSQ humor styles and the HBQD-RF styles of humorous conduct. As this only partly confirms Prediction 6, future studies on the relationships between the HSQ and the styles of humorous conduct are warranted. The variance explained in the ten unipolar styles of humorous conduct by the four HSQ scales was also large (answering Research Question 3), with the strongest overlaps occurring for the socially warm and cold (HSQ affiliative and self-enhancing), mean-spirited and earthy (HSQ aggressive and self-defeating), and competent style of humorous conduct (all four HSQ scales). The styles of everyday humorous conduct reflective, inept, and benign were least represented in the HSQ, and the four HSQ scales explained up to 60% of their reliable variance. In turn, the HBQD styles of humorous conduct explained large amounts of variance in the HSQ scales, with the largest effect occurring for the HSQ affiliative scale (answering Research Question 4).

The descriptions of the eight comic styles according to Schmidt-Hidding (1963), by contrast, showed lower correlations to the HSQ scales, which is also due to the fact that they were assessed with single ratings. The five predicted relationships were confirmed with medium to large effects; that is, affiliative went along with fun, aggressive went along with satire, sarcasm, and cynicism, and self-enhancing with humor (small to medium effects). In addition, the aggressive humor style also correlated with wit and irony and negatively with (benevolent) humor. This could indicate that people high in the aggressive humor style might have a larger repertoire of different ways of showing humor. Another explanation could be that many comic styles contain an aggressive element. This makes sense as *disparagement humor* is often considered an important element in humor (for a theoretical and empirical overview, see Ferguson & Ford, 2008).

This explanation might have two consequences: First, some of the comic styles (especially satire, sarcasm, and cynicism) were rather similar in self-reports (correlations between the three ratings ranged from .48 to .58, indicating large overlaps), although their construct descriptions differed from one another. Although this indicates at least 23.0–33.6% shared variance between the three ratings, it cannot be evaluated how much reliable variance the ratings share. Second, the HSQ aggressive scale is sensitive to the mocking and teasing element in these comic styles, but not to the nuances in which these comic styles differ (e.g., cynicism representing a general destructive attitude, while satire aims at correcting societal problems and wrongdoings of others). Further research is needed to investigate the aggressive elements in the comic styles further and to delineate their usefulness as individual difference variables in humor research. If a separation of these comic styles (e.g., satire, sarcasm, cynicism) is possible, maybe a facet model with different mockery components would be favorable.

As expected, the self-enhancing and self-defeating humor styles had small correlations to some of the comic styles. In turn, the comic styles nonsense and irony only had small correlations to the HSQ scales, while the correlations were higher than expected for wit and satire (both correlating with the HSQ aggressive scale). Thus, Prediction 7 can only be partially confirmed.

Regarding the variance the four HSQ scales could explain in the comic styles (Research Question 5), the values were large. The smallest amounts of variance were explained in irony and nonsense. These two comic styles entail specific linguistic techniques (saying the opposite of what is meant) and specific humor contents (nonsensical play on words and the play with sense and nonsense) that are not covered in the HSQ humor styles. Conversely, the eight comic styles explained a medium to large amount of variance in the HSQ self-enhancing and self-defeating scales, while the effects were large for affiliative and aggressive (Research Question 6). This shows that the aspects of coping humor and humor ineptness were less represented in the comic styles.

In general, the styles of humorous conduct and comic styles showed many overlaps with the HSQ scales, the former mainly with the affiliative and aggressive humor styles and the latter especially with the aggressive humor style. Yet it is underscored that they measure different humor aspects than the HSQ does, despite similar labels. This also fits to the theoretical distinctions of the definitions and the differential usage of the term styles. The HSQ humor styles focus on evaluations of different uses of humor, while the HBQD-RF and the comic styles focus more on humor behaviors and different ways they can be conducted, converging more with the approach of stylistic traits. These style approaches are thus non-redundant and each should be studied in their own right.

What do the findings of the present study imply for future research in the area of styles of humor and HSQ humor styles in particular? First, the successful adaptation of the HSQ into German and the investigation of its psychometric properties and norms allow unifying research efforts in German-speaking countries. Second, establishing strong factorial invariance and similar means across two countries provide a fruitful basis for comparative international studies, extending the present findings to further languages and cultural areas. Third, the lack of support for strong factorial invariance for gender and three age groups and the mean differences in these demographic groups open up a new research venue: Why do men differ from women and why do adolescents differ from adults? Do they evaluate humor differently; for example, do men regard aggressive humor styles as less detrimental to others than women do? Or do they use humor for different purposes? These questions and others would provide insights into our understanding of the HSQ humor styles in general and in prevalent demographic groups.

Fourth, the inclusion of the other two approaches to styles of humor theoretically and empirically complements studies of the HSQ by focusing more on humor behaviors and how they are performed. Future studies could investigate whether the styles of everyday humorous conduct and the comic styles do also contribute to psychosocial well-being, especially beyond the HSQ scales. As the HSQ was proposed to be comprehensive in terms of humor and well-being, this should not be the case, yet an empirical test of this proposition has yet to be provided. This would further strengthen the HSQ as a comprehensive measure in this area, or it could suggest possible extensions of the HSQ.

### Limitations

Although the sample was large, it was not representative of the German-speaking population, especially in terms of age and education. The psychometric properties of the HSQ might differ in people with lower levels of education or in older people, so this remains to be tested in future research. Second, the comic styles were measured with one item each; thus they might have low reliability, limiting the correlations that can be obtained with other measures. Future studies should employ several items to measure each of the comic styles described by Schmidt-Hidding (1963) as an individual-difference variable.

### Conclusions

The present study examined the psychometric properties of the German version of the HSQ in a large sample of adults, and presents its norms as a reference for future studies using this adaptation. Overall, the psychometric properties of the HSQ were similar to the original paper (Martin et al., 2003) and better than in many other translations (e.g., Bilge & Saltuk, 2007; Chen & Martin, 2007; Kazarian & Martin, 2006; Taher et al., 2008), supporting that it can be used meaningfully in German-speaking countries (at least Germany and Switzerland). The reliability of the four HSQ scales was supported in the total sample as well as in the subgroups, with the aggressive scale showing the lowest reliabilities. Weak factorial invariance for the four-factor structure of the HSQ was supported across gender and age groups, while strong factorial invariance was supported across Switzerland and Germany. This supports the feasibility of interpreting differences in the four HSQ factors in these demographic subgroups as the items form at least the same latent structure. The empirical convergence of the HSQ scales with 10 styles of humorous conduct and eight comic styles was mixed, with some styles largely converging with the HSQ, while others were rather independent of it. This is line with the theoretical distinctions of stylistic traits and the approaches to styles in the three humor concepts, underscoring that the usage of similar labels does not automatically imply that the constructs are interchangeable as well.
